# Supplementary material for: Effects of salbutamol on the kinetics of sevoflurane and the occurrence of early postoperative pulmonary complications in patients with mild-to-moderate chronic obstructive pulmonary disease: A randomized controlled study
Source: PLoS One. 2021 May 20;16(5):e0251795. doi: 10.1371/journal.pone.0251795 (PMC8136676; doi:10.1371/journal.pone.0251795)
Supplement: S1 Table — (DOCX) [file pone.0251795.s002.docx]

S1 table The profiles of the wash-in of sevoflurane within 15 minutes after initiation

|  | 1 min | 2 min | 3 min | 4 min | 5 min | 7 min | 10 min | 15 min |
| --- | --- | --- | --- | --- | --- | --- | --- | --- |
| Salbutamol | 0.41±0.05 | 0.49±0.10 | 0.55±0.09 | 0.65±0.09 | 0.71±0.10 | 0.82±0.11 | 0.84±0.09 | 0.86±0.08 |
| Control | 0.39±0.06 | 0.45±0.08 | 0.47±0.10 | 0.50±0.12 | 0.58±0.08 | 0.71±0.11 | 0.75±0.06 | 0.81±0.10 |
| *P* value | 0.102 | 0.085 | 0.035 | 0.002 | 0.042 | 0.041 | 0.044 | 0.143 |
