# Supplementary material for: Effects of salbutamol on the kinetics of sevoflurane and the occurrence of early postoperative pulmonary complications in patients with mild-to-moderate chronic obstructive pulmonary disease: A randomized controlled study
Source: PLoS One. 2021 May 20;16(5):e0251795. doi: 10.1371/journal.pone.0251795 (PMC8136676; doi:10.1371/journal.pone.0251795)
Supplement: S2 Table — (DOCX) [file pone.0251795.s003.docx]

S2 table The profiles of the wash-out of sevoflurane after closing vaporizer

|  | 1 min | 2 min | 3 min | 4 min | 5 min | 7 min | 10 min | 15 min |
| --- | --- | --- | --- | --- | --- | --- | --- | --- |
| Salbutamol | 0.97±0.05 | 0.88±0.10 | 0.81±0.10 | 0.0.72±0.07 | 0.66±0.09 | 0.57±0.09 | 0.34±0.06 | 0.23±0.05 |
| Control | 0.94±0.06 | 0.80±0.07 | 0.80±0.10 | 0.50±0.10 | 064±0.07 | 0.53±0.10 | 0.32±0.05 | 0.21±0.0.06 |
| *P* value | 0.971 | 0.672 | 0.132 | 0.082 | 0.144 | 0.092 | 0.178 | 0.231 |
