# Supplementary material for: Effects of salbutamol on the kinetics of sevoflurane and the occurrence of early postoperative pulmonary complications in patients with mild-to-moderate chronic obstructive pulmonary disease: A randomized controlled study
Source: PLoS One. 2021 May 20;16(5):e0251795. doi: 10.1371/journal.pone.0251795 (PMC8136676; doi:10.1371/journal.pone.0251795)
Supplement: S1 File — (DOCX) [file pone.0251795.s005.docx]

The clinical research protocol

1. **Purposes:**

We speculated that salbutamol aerosol used preoperatively ameliorates the bronchoconstriction, improves lung volume and gas distribution, eventually affects the alveolar-capillary interface.

To investigate the effects of salbutamol used preoperatively on the kinetics of volatile sevoflurane(wash-in) and the wash out curves after closing vaporizer, and the recovery profile when stay in the PACU, and whether this would reduce the occurrence of early postoperative pulmonary complications (the first 7days) in patients with mild to moderate chronic obstructive pulmonary disease.

**2.Background**

Chronic obstructive pulmonary disease (COPD) is a preventable and treatable disease characterized by airflow limitation, which is not completely reversible and develops progressively. A survey of 102230 adults in rural areas of northern and central China showed that COPD accounted for about 3% the population aged 15 and over, and the prevalence rate COPD people aged 40 and over was 8.2 %.COPD results mainly in excessive pulmonary inflation, limited airflow and abnormal gas exchange. Lung function examination showed total lung volume (TLC), functional residual volume (FRC), increased residual volume (RV) and vital capacity (VC), forced vital capacity (FVC), forced expiratory volume (FEV) in the first second_._

Because of the above pathophysiological changes in the respiratory system in COPD patients, COPD patients undergo a slow increase in intra-alveolar anesthetic concentrations during inhalation anesthesia, decreased uptake through alveolar capillary membrane transmembrane, causing a low concentration of "effector sites "(e.g. brain) drugs. The anesthetic depth is not easy to deepen or to reach the predetermined level within a certain period of time. Moreover, during the anaesthesia recovery phase, the concentration gradient between capillaries and alveoli decreases due to airflow obstruction. The patient had a delayed recovery, poor recovery quality (e.g., irritability after waking) or delayed respiratory depression, even life-threatening events occur.

Salbutamol selectively stimulates the β_2_ of bronchial smooth muscle receptor, mainly used to prevent and treat bronchial asthma or asthmatic bronchitis. Salbutamol in the treatment of COPD patients, can improve lung function, improve exercise tolerance, improve the quality of life has been verified. Recent studies suggest β_2_ receptor agonists increase FEV_1_To FVC, reduce RV 、FRC and improve airflow patterns in COPD patients.

This study was to explore whether salbutamol pretreatment can change COPD respiratory mechanics and lung volume, and affect the wash-in, uptake and elimination of sevoflurane. Whether it is beneficial to improve postoperative pulmonary function.

**3. Research protocol**

**3.1 Types of research**

Prospective, randomized, controlled, clinical studies

**Primary outcome**

Ratio of inhaled sevoflurane concentration (In_sevo_) to end-expiratory sevoflurane concentration (Et_sevo_ )，namely the F_A_/F_I_ fraction.

**Secondary endpoints**

(1) The time of opening eyes, extubation and oral birthday of patients in anesthesia recovery room.

(2) Number and incidence of pulmonary complications within 7 days after operation.

3.2 **Research methodology**

**3.2.1 Research site**

Shaoxing People's Hospital

**3.2.2 Research participants**

1. patients with mild to moderate COPD were recruited in the study.

(1)Inclusion criteria:

ASA Ⅰ~ II grade ;65~75 years old;

long-term smoking history or previous confirmed chronic bronchitis, emphysema ;

Pulmonary function test was performed before surgery and the severity of classification was obtained (mild to moderate COPD);

Body mass index18~30 kg/m^2^;

No upper respiratory infection within 2 weeks before surgery;

Stable COPD patients

(2) Exclusion criteria:

Allergic to β_2_ receptor agonists, alcohol and freon;

pulmonary arterial hypertension (PAP ﹥50 mmHg at rest);

cardiac insufficiency or heart failure;

patients refused to cooperate;

renal insufficiency (BUN﹥10 mmol/L ,Cr﹥1.5mg/dl）。

(3) Exclusion criteria:

patient withdrawal;

incomplete data;

loss of follow-up ;

adverse events

**3.2.3 Main research methods**

Salbutamol (40 vial) and its similar in appearance placebo (40 vial, normal saline) were used in this study. The computer-generated random sequence numbers were stored in an opaque envelope. The investigators who were responsible for assessing the primary endpoints, as well as the anesthesiologists, postoperative care unit nursing staff, and variable assessors, were blinded to study group assignment.

Before operation, signed informed consent was obtained, no preoperative medication was administered , no food for 10 h and 4 h for drinking before operation.

*Pre-anesthesia preparation:*

Routine monitoring was initiated, the peripheral vein was cannulated, and sodium lactate Ringer's solution 500 mL for volume expansion;

Radial artery catheterization was performed to monitor invasive blood pressure;

Give salbutamol reagent oral spray 200 μg (2 spray) and record reagent number;

No drugs (antibiotics, hormones, etc.) were given before induction, and no test dose was injected if epidural catheter was retained.

*BIS monitoring:*

Clean the skin repeatedly with alcohol cotton balls and use BIS after drying^XP^Dedicated four-conductor electrode (BIS-sensor) chip^XP^), Electrode 1 is facing the eyebrow (about 5 cm from the nose root), Electrode 2 is located between 1 and 4 above the brow, The lower edge of electrode 4 is aligned with the right eyebrow arch, Electrode 3 is aligned with the center of the eye.

*Anesthesia induction:*

Salbutamol aerosol spray or placebo was administered through inhalation 30 min before anesthesia induction. Oxygen flow rate was set to 8 L/min before oxygenation. Fentanyl 3.0 μg/kg was injected intravenously slowly, Propofol 1.5~2.0 mg/kg rocurium 0.9 mg/kg；Tracheal tube was fixed after successful tracheal intubation (female ID 7.0, male ID 8.0), then mechanical control ventilation was initiated, adjusting tidal volume 8~10 ml/kg respiratory rate 10 bpm, I/E ratio 1:2, to keep EtCO_2_ within 30 to 45 mmHg .When the hemodynamics is stable for 4~5 min, the sevoflurane volatile vaporizer was opened (2% sevoflurane was used to prime the respiration circuit to ensure the uniform filling of the circuit), the inhalation concentration was 2% and the oxygen flow rate was 2 L·min^-1^. The concentration of sevoflurane was collected at one end of the Y interface of the endotracheal tube through sidestremline method. During the wash -out period, the oxygen flow rate was 4 L/min .

*Anaesthesia maintenance:*

No exogenous stimulation (including changing the position) and no anesthetic drugs were added during observation. Ephedrine 5~10 were injected intravenously (MAP ﹤50 mmHg) during observation. Atropine 0.25~0.5 mg was given when the heart rate (﹤45 times/min) .Salbutamol 200 or placebo was given through the respiratory circuit 30 min before the completion of surgery, The vaporizer was closed 30 minutes before the last skin suture was placed, with fresh gas flow set at 4L/min. Following this, end-tidal samples were collected from first breaths at 1, 2, 3, 4, 5, 7, 10 and 15 min after discontinuation of its administration.

*Observation variables*

End-tidal samples were collected from first breaths at 1, 2, 3, 4, 5, 7, 10 and 15 min, and then the surgery began. The hemodynamic parameters and airway pressure data in the corresponding time points.

End-tidal samples were collected from first breaths at 1, 2, 3, 4, 5, 7, 10 and 15 min after discontinuation of its administration. The hemodynamic parameters and airway pressure data in the corresponding time points.

Arterial blood samples were extracted from the patients before induction (0 min), then at 30 min and 60 min after initiation of the operation.

Observe the occurrence of pulmonary complications in all patients within 7 days after operation.

*Statistical analysis*

After all the tests, according to the random number, the subjects were divided into salbutamol group and control group for statistical analysis. Stata7.0 software package for statistical analysis, The mean ± standard deviation (mean±SD) are used for all measurements, A complete randomized t test was used to test the differences between groups, Analysis of variance (ANOVA,) within groups S-N-K methods), Comparison of counting data using chi-square test or Fisher exact probability calculation. P ﹤0.05, the difference was statistically significant.

**4. The flow chart**

80 patients with COPD were enrolled to the study

Salbutamol aerosol 200μg or placebo

Anestheisa induction

After stable hemodynamic maintained, the vaporizer opened

Insevo and Etsevo 1min,2min,3min,5min,7min,10min and 15 min after open vaporizer, and airway pressure

Surgery and anesthesia maintenance

Salbutamol aerosol 200μg

or placebo via circuit

Insevo and Etsevo 1min,2min,3min,5min,7min,10min and 15 min after closing vaporizer, and airway pressure

Time of open eyes, extubation, date of birth,etc.

Recovery in PACU

Observe the PPC within

7days after surgery

Unblinding when finishing the study

Salbutamol group

Control group

Statistical analysis

**5.Appendix**

The diagnosis criteria of COPD patients: COPD diagnosis is mainly based on the comprehensive analysis of high risk history, clinical symptoms, signs and lung function of smoking. Long history of cough and expectoration; barrel chest, chest percussion, prolonged expiratory time; chest radiographs showing signs of emphysema; incomplete reversible airflow limitation as a necessary condition for COPD diagnosis ;70% FVC < and FEV after inhalation of bronchiectasis_l_<80% of the predicted value, it can be determined as incomplete reversible airflow limitation. Bronchiectasis test positive: △FEV_1_/FEV_l_≥12 % and △FEV_1_ ≥200ml.

Criteria for determining PPC: Components include pulmonary inflammation, respiratory failure, pleural effusion, atelectasis, pneumothorax, bronchospasm, aspiration pneumonia

Definitions of postoperative pulmonary complications

*Respiratory infection*

Treatment with antibiotics for a respiratory infection,plus at least one of the following criteria：new or changed sputum,new or

changed lung opacities,fever,and leukocyte count >12,000/ mm 3 per cent

*Respiratory failure*

Postoperative PaO 2<60 mmHg on room air,a ratio of PaO 2to inspired oxygen fraction Postoperative PaO 2<300, or SaO 2Postoperative PaO 2<90 per cent

therapy

*Pleural effusion*

Chest radiograph demonstrating blunting of the costophrenic angle,evidence of displacement of adjacent anatomical structures,or

(in supine position) a hazy opacity in one hemithorax with preserved variable shadows

*Atelectasis*

Collapse of the alveoli,lung opacification with shift of the mediastinum,hilum,or hemidiaphragm toward the affected area,and

compensatory overinflation in the adjacent nonatelectatic lung

*Pneumothorax*

(A collection of air in the pleural space)

*Bronchospasm*

Newly detected expiratory wheezing treated with bronchodilators

*Aspiration pneumonitis*

Acute lung injury after the inhalation of regurgitated gastric contents

PaO2：partial pressure of oxygen in arterial blood;SaO 2：arterial oxyhemoglobin saturation.

**6. References**

(1) Downs CA,Appel SA.Chronic obstructive pulmonary disease：Diagnosis and management.Am Acad Nurse Pract ,2007,19：126-132.

(2) Burrowes KS,De Backer J,Smallwood R,et al.Interface Focus,2013,3(2)：20120057.[Multi-scale computational models of the airways to unravel the pathophysiological mechanisms in asthma and chronic obstructive pulmonary disease (AirPROM).](http://www.ncbi.nlm.nih.gov/pubmed/24427517)

(3) Zoeckler N,Kenn K,Kuehl K,et al.[Illness perceptions predict exercise capacity and psychological well-being after pulmonary rehabilitation in COPD patients.](http://www.ncbi.nlm.nih.gov/pubmed/24439691)

J Psychosom Res,2014,76(2)：146-151.

(4) Wu Y,Liu F,Tang H,et al.Anesth Analg,2013,117(2)：507-513.[The analgesic efficacy of subcostal transversus abdominis plane block compared withthoracic epidural analgesia and intravenous opioid analgesia after radical gastrectomy.](http://www.ncbi.nlm.nih.gov/pubmed/23744953)

(5) Agzarian J,Miller JD,Kosa SD,et al.Ann Thorac Surg,2013,96(4):1217-1222...[Long-term survival analysis of the Canadian Lung Volume Reduction Surgery trial.](http://www.ncbi.nlm.nih.gov/pubmed/23895890)

(6) Peduto VA ,Mezzetti D ,Properzi M ,et al.Sevoflurane provides better recovery than propofol plus fentanyl in anaesthesia for day-care surgery.Eur J Anaesthesiol ,2008,17：138-143.

(7) Hovens IB,Schoemaker RG,van der Zee EA,et al.Brain Behav Immun,2012,26(7):1169-1179...[Thinking through postoperative cognitive dysfunction：How to bridge the gap between clinical and pre-clinical perspectives.](http://www.ncbi.nlm.nih.gov/pubmed/22728316)

(8) Peyton PJ,Fortuin M,Robinson GB,et al.The rate of alveolar-capillary uptake of sevoflurane and nitrous oxide following anaesthetic induction .Anaesthesia,2008,63：358-363.

(9) Zulkarneev R,Zagidullin N,Abdrahmanova G,et al.Pharmaceuticals (Basel),2012,5(4):[Ivabradine prevents heart rate acceleration in patients with chronic obstructive pulmonary disease and coronary heart disease after salbutamol inhalation.](http://www.ncbi.nlm.nih.gov/pubmed/24281409)

398-404.

(10) Tantucci C ,Duguet A ,Similowski T ,et al.Effect of salbutamol on dynamic hyperinflation in chronic obstructive pulmonary disease patients.Eur Respir J ,2010,12：799-804.

(11) Ross Kennedy R,French RA,Spencer C.Predictive Accuracy of a Model of Volatile Anesthetic Uptake.Anesth Analg 2002;95：1616–1621.

[Dose-response curve to salbutamol during acute and chronic treatment with formoterol inCOPD.](http://www.ncbi.nlm.nih.gov/pubmed/21857779)(12) La Piana GE,Corda L,Bertella E,et al.I nt J Chron Obstruct Pulmon Dis,2011,6：399-405.

(13) Cazzola M,Rogliani P,Ruggeri P,Set al.Respir Med.2013Jun;107(6)：848-853.[Chronic treatment with indacaterol and airway response to salbutamol in stable COPD.](http://www.ncbi.nlm.nih.gov/pubmed/23490225)

(14) De Backer LA,Vos WG,Salgado R,et al. [Functional imaging using computer methods to compare the effect of salbutamol and ipratropium bromide in patient-specific airway models of COPD.](http://www.ncbi.nlm.nih.gov/pubmed/22162649) Int J Chron Obstruct Pulmon Dis,2011;6(2):637-646.

(15) Patman S.Preoperative physiotherapy education prevented postoperative pulmonary complications following open upper abdominal surgery.BMJ Evid Based Med.2019;24(2):74-75...

(16) Nijbroek SG,Schultz MJ,Hemmes SNT.Prediction of postoperative pulmonary complications.Curr Opin Anaesthesiol.2019;32(3):443-451...

(17) Miskovic A,Lumb AB.Postoperative pulmonary complications.Br J Anaesth.2017 1;118(3)：317-334.
