# Supplementary material for: Effects of salbutamol on the kinetics of sevoflurane and the occurrence of early postoperative pulmonary complications in patients with mild-to-moderate chronic obstructive pulmonary disease: A randomized controlled study
Source: PLoS One. 2021 May 20;16(5):e0251795. doi: 10.1371/journal.pone.0251795 (PMC8136676; doi:10.1371/journal.pone.0251795)
Supplement: S2 File — (PDF) [file pone.0251795.s006.pdf]

	level	control	salbutamol	p
n		33	30	
Age (mean (SD))		65.42 (7.46)	67.70 (6.49)	0.641
Body_Weight (mean (SD))		62.48 (8.91)	63.53 (10.52)	0.712
Gender (%)	F	10 (30.3)	0 (0.0)	0.461
	M	23 (69.7)	30 (100.0)	
ASA (%)	1	1 (3.0)	3 (10.0)	0.235
	2	29 (87.9)	25 (83.3)	
	3	3 (9.1)	2 (6.7)	
NYHA (%)	1	4 (12.1)	12 (40.0)	0.058
	2	25 (75.8)	15 (50.0)	
	3	4 (12.1)	3 (10.0)	
Hypertension (%)	No	17 (51.5)	15 (50.0)	0.678
	Yes	16 (48.5)	15 (50.0)	
Cerebrovascular (%)	No	29 (87.9)	29 (96.7)	0.411
	Yes	4 (12.1)	1 (3.3)	
Respiratory (%)	bronchitis	1 (3.0)	0 (0.0)	0.625
	COPD	6 (18.2)	6 (20.0)	
	emphysema	26 (78.8)	24 (80.0)	
Urological (%)	No	32 (97.0)	28 (93.3)	0.933
	Yes	1 (3.0)	2 (6.7)	
Endocrine (%)	No	28 (84.8)	29 (96.7)	0.243
	Yes	5 (15.2)	1 (3.3)	
Wash_in_1min (mean (SD))		0.43 (0.09)	0.45 (0.12)	0.53
Wash_out_1min (mean (SD))		1.00 (0.37)	0.92 (0.29)	0.069
Wash_in_2min (mean (SD))		0.56 (0.09)	0.54 (0.12)	0.338
Wash_out_2min (mean (SD))		0.92 (0.31)	0.83 (0.24)	0.013
Wash_in_3min (mean (SD))		0.65 (0.11)	0.74 (0.23)	0.041
Wash_out_3min (mean (SD))		0.86 (0.30)	0.75 (0.23)	0.054

Wash_in_4min (mean (SD))		0.68 (0.14)	0.79 (0.23)	0.038
Wash_out_4min (mean (SD))		0.78 (0.29)	0.70 (0.22)	0.077
Wash_in_5min (mean (SD))		0.86 (0.18)	0.98 (0.21)	0.005
Wash_out_5min (mean (SD))		0.63 (0.26)	0.64 (0.21)	0.072
Wash_ in_7min (mean (SD))		0.97(0.20)	1.08 (0.23)	0.007
Wash_out_7min (mean (SD))		0.52 (0.22)	0.55 (0.22)	0.071
Wash_ in_10min (mean (SD))		1.11 (0.23)	1.31 (0.26)	0.021
Wash_out_10min (mean (SD))		0.58 (0.20)	0.42 (0.22)	0.004
Wash_in_15min (mean (SD))		1.40 (0.24)	1.30 (0.26)	0.106
Wash_out_15min (mean (SD))		0.41 (0.18)	0.39 (0.19)	0.054
Wash_ in_Ppeak (mean (SD))		18.33 (3.65)	16.80 (2.38)	0.056
Wash_out_Ppeak (mean (SD))		19.70 (3.57)	17.83 (2.89)	0.027
Wash_in_Pmean (mean (SD))		13.36 (3.75)	9.70 (2.79)	<0.001
Wash_out_Pmean (mean (SD))		14.76 (3.25)	11.40 (3.64)	<0.001
Squeeze_hand (mean (SD))		30.61 (12.00)	36.20 (12.20)	0.072
Open_eye (mean (SD))		28.82 (12.25)	26.23 (13.11)	0.104
Date_of_birth (mean (SD))		33.70 (11.62)	38.50 (11.84)	0.11
Discharge_ from_PACU (mean (SD))		61.58 (24.22)	64.30 (23.03)	0.737
ICU_admission (%)	No	28 (84.8)	28 (93.3)	0.504
	Yes	5 (15.2)	2 (6.7)	
PaO2/FiO2_baseline (mean (SD))		269.09 (21.34)	271.50 (20.02)	0.132
PaO2/FiO2_30min (mean (SD))		291.45 (29.57)	320.30 (17.62)	0.033
PaO2/FiO2_60min (mean (SD))		309.21 (30.53)	327.83 (32.30)	0.003
VD/VT_baseline (mean (SD))		0.254 (0.052)	0.262 (0.064)	0.245

**VD/VT_30min (mean (SD))**		0.263 (0.06)	0.205 (0.064)	0.042
**VD/VT_60min (mean (SD))**		0.248 (0.055)	0.196 (0.051)	0.007
**Hypoxia (%)**	No	31 (93.9)	30 (100.0)	0.515
	Yes	2 (6.1)	0 (0.0)	
**Pneumonia (%)**	No	30 (90.9)	28 (93.3)	1
	Yes	3 (9.1)	2 (6.7)	
**Pneumothorax (%)**	No	33 (100.0)	30 (100.0)	NA
**Atelectasis (%)**	No	31 (93.9)	29 (96.7)	1
	Yes	2 (6.1)	1 (3.3)	
**Bronchospasm (%)**	No	25 (75.8)	27 (90.0)	0.023
	Yes	8 (24.2)	3 (10.0)	
Pulmonary infiltrate(%)	No	24	28	0.017
	Yes	9	2	
