# Supplementary material for: Effects of salbutamol on the kinetics of sevoflurane and the occurrence of early postoperative pulmonary complications in patients with mild-to-moderate chronic obstructive pulmonary disease: A randomized controlled study
Source: PLoS One. 2021 May 20;16(5):e0251795. doi: 10.1371/journal.pone.0251795 (PMC8136676; doi:10.1371/journal.pone.0251795)
Supplement: S3 File — (DOCX) [file pone.0251795.s007.docx]

临床研究实行方案

沙丁胺醇对慢阻肺疾病全麻患者七氟烷药代动力学、苏醒质量和早期肺部并发症的影响

1. **研究目的**

探讨术前和术中使用沙丁胺醇对老年慢阻肺疾病全麻患者七氟烷的摄取和排除的影响，观察是否对术后苏醒和早期肺部并发症的发生产生影响。

**2.研究背景**

慢性阻塞性肺疾病(chronic obstructive pulmonary disease，COPD)是一种以气流受限为特征的可以预防和可治疗的疾病，气流受限不完全可逆，呈进行性发展^[1,2]^。中国北部及中部地区农村102230成年人群抽样调查显示，COPD 约占15 岁以上人群的3%，40岁以上人群COPD患病率为8.2%。COPD主要引起肺过度充气、气流受限和气体交换异常，肺功能检查表现为肺总量(TLC)、功能残气量(FRC)、残气量(RV)增高和肺活量(VC)、用力肺活量(FVC)、第1秒用力呼气量(FEV_1_)下降^[3]^。而老年人的呼吸系统解剖学变异主要表现为胸壁弹性差、气道狭窄、肺泡表面积减少、弹性纤维改变，使得老年人肺顺应性随年龄增高而下降，其VC下降, TLC、FRC、RV及死腔率(V_D_/V_T_)增高，肺泡弥散能力下降，换气功能下降，进而表现为通气/血流（V/Q）比例失调。老年COPD患者因年龄本身引起的生理机能退变和COPD造成的病理生理改变，呼吸系统改变更加显著，通气功能明显下降，肺泡弥散能力下降，气体交换功能异常。因此，老年COPD患者多有阻塞性通气功能障碍、低氧血症，甚至肺外的其它不良反应。因COPD患者存在呼吸系统的上述病理生理改变，COPD患者在实施外科手术吸入麻醉时会出现肺泡内麻醉药物的浓度上升缓慢，吸入麻醉药单位时间内经肺泡－毛细血管膜跨膜“摄取”减少，造成“效应部位”（如脑）药物浓度较低，导致麻醉深度不容易加深或者在一定时间内麻醉深度不容易达到预定的水平。然而，在麻醉苏醒阶段，因气流受阻毛细血管与肺泡间药物浓度梯度减小，弥散速率减慢，患者出现苏醒延迟，苏醒质量欠佳（如醒后烦躁）或出现延迟性呼吸抑制，甚至危及生命事件发生。因此，如果能够对此类患者围手术期预见性地给予某种支气管扩张药物干预处理，将有利于麻醉加深阶段（即吸入麻醉药的“洗入”）药物浓度快速升高，麻醉深度短时间内维持在一定水平，苏醒阶段缩短苏醒过程，减少药物残留，使围术期的麻醉管理更加方便，同时节省麻醉药物的用量，使患者手术结束后主观感觉良好，减少麻醉并发症的发生。为解决上述临床问题，本项目拟采用一种支气管扩张剂――沙丁胺醇在麻醉前30 min和苏醒前30 min通过定量吸入装置给予进行吸入麻醉的COPD患者（试验组和对照组），观察和测定不同阶段和不同时间点吸入麻醉药物的浓度，记录患者在不同时间的反应，最后比较两组之间的差别，给围手术期管理带来方便，减少并发症，给患者提供舒适化医疗，提供更加安全的保障。

随着社会的发展和生活质量的改善，人均寿命不断的延长，全世界都将面临人口老龄化的问题。据统计，65岁以上的老年人中，在人生中约半数至少经历一次手术治疗。由于生理机能在各方面都出现了退行性改变，老年患者围术期的并发症和死亡率显著高于青壮年。因此，如何降低老年患者的手术麻醉并发症，保证患者的安全成为我们日常工作的巨大挑战。

由于七氟烷的药理学特点和药理学作用，现在已经广泛应用于临床麻醉；并且老年COPD患者接受外科手术（如肺减容术和肺癌手术等）时采用吸入麻醉的人数也逐渐增多。然而，老年患者和老年COPD患者因死腔量和FRC增加以及动态性肺充气，采用七氟烷麻醉时麻醉诱导阶段往往出现呼气末麻醉气体浓度并不能反映实际的麻醉深度^[4]^和麻醉深度不容易达到预定水平，麻醉苏醒阶段因气体陷闭则会出现吸入麻醉药排除缓慢，导致苏醒延迟和延时拔管，因药物残余拔管后可能出现呼吸抑制甚至再次气管插管。

有研究表明七氟烷麻醉后患者苏醒时间和苏醒质量均优于异丙酚并且在某些患者七氟烷麻醉后脑内氧分压较异丙酚高，前者术后神志功能障碍的发生率较低^[7]^，因此目前更倾向于七氟烷实施麻醉，尤其是高龄患者。因此，若采用预处理能够改变老年患者和老年COPD患者气道相关因素，进而加快吸入麻醉药向肺泡内“洗入”和跨肺泡－毛细血管膜的摄取，使得麻醉深度在一定时间达到预定水平；而在麻醉苏醒阶段可以加速吸入麻醉药经肺部快速“洗出”，患者术后可以快速苏醒，提高苏醒质量，减少延迟性呼吸抑制，将会对此类患者围手术期麻醉管理产生积极影响。

沙丁胺醇能选择性激动支气管平滑肌的β_2_受体，是一种较强的支气管扩张剂，主要用于预防和治疗支气管哮喘或喘息型支气管炎等伴有支气管痉挛（喘鸣）的呼吸道疾病，改善患者的通气功能，其使用的安全性已经过多年的临床验证。沙丁胺醇用于COPD患者的治疗，可以改善肺功能，提高运动耐量，改善生活质量方面的效果已经得到验证。近期研究表明β_2_受体激动剂能够增加FEV_1_、FVC，降低RV、FRC和改善COPD患者气流输送形式；目前有3位研究者分别对肺功能正常老年患者七氟烷的麻醉诱导、诱导后摄取进行研究，2009年项目申请者对COPD患者七氟烷摄取进行了部分研究，部分研究^[13,14]^提示沙丁胺醇预处理后不能加快肺功能正常老年患者七氟烷跨肺泡摄取，而麻醉前吸入沙丁胺醇能否改善老年COPD患者肺功能，扩张支气管和终末呼吸性细支气管，改变FRC、RV及V_D_/V_T_比例，增加麻醉气体有效输送和吸入麻醉药经毛细血管-肺泡膜摄取的有效单元，进而加快七氟烷的摄取和缩短麻醉达到一定深度所需时间，尚未见报道。

本研究结合COPD患者呼吸功能改变，探讨沙丁胺醇预处理后能否改变COPD呼吸力学和肺容量，对七氟烷洗入、摄取、消除产生影响，并最终达到在麻醉加深阶段（即吸入麻醉药的“洗入”）药物浓度能快速升高，麻醉深度短时间内处于一定的水平；苏醒阶段能快速清醒，药物残留较少，使COPD患者围手术期管理更加方便，并减少不良事件的发生（术后早期肺部并发症），解决临床麻醉中的实际问题。

沙丁胺醇改善呼吸动力学，可能提高氧合，增加大脑和心脏的氧供，是否有利于改善术后认知功能障碍、降低心血管事件的发生，减少住院日和住院费用消耗也是今后需要进一步探讨之处。

3．研究方案

3.1研究类型和目标

前瞻、随机、对照、临床研究

3.1.1主要指标

吸入七氟烷浓度(Insevo)%和呼气末七氟烷浓度(Etsevo)%之比 (F_A_/F_I_)。

3.1.2 次要研究指标

(1)麻醉恢复室患者睁眼、拔管和口述生日等时间。

(2)术后早期7天内患者肺部并发症发生例数和发生率。

3.2研究方法

3.2.1研究现场

绍兴市人民医院

3.2.2研究对象

本次实验纳入组患者择期进行外科手术COPD患者78例。

(1)纳入标准：

ASAⅠ～Ⅱ级；65～75岁；长期吸烟史或曾经确诊慢性支气管炎、肺气肿及COPD；手术前进行肺功能测定报告和分级（选择轻度和中度COPD病例）；BMI18～30 kg·m^-2^；2周内无上感史；COPD处于稳定或缓解期严格执行纳入标准和排除标准以选择合适的研究病例。

(2)排除标准：

β_2_受体激动剂、酒精、氟里昂过敏者；肺动脉高压（PAP﹥50 mmHg）；心功能不全或心力衰竭；患者拒绝合作； 肾脏功能不全（BUN﹥10 mmol·L^-1^, Cr﹥1.5 mg·dl^-1^）。

研究获得医院伦理委员会审核和批准，研究时每例患者均签署知情同意书。

(3)剔除标准： 患者中途退出，数据不全，失去随访和不良事件等。

2.3主要研究方法

将40支沙丁胺醇样品试剂和40支空白试剂（成分为生理盐水）混合，由电脑生成随机数字进行编号，根据编号顺序使用研究药物。术前晚常规访视病例，签署知情同意书，无术前用药，术前禁食10 h以上，禁饮4 h。

（1）麻醉前准备：

①患者入室后，常规监护，开放外周静脉，诱导前预先给予乳酸钠林格氏液500 mL扩容；

②行桡动脉置管，监测有创血压；

③给予沙丁胺醇试剂口腔喷雾200 μg（2喷），并记录试剂编号；

④诱导前不给与任何药物（抗生素、激素等），若留置硬膜外导管，则不加试验剂量。

⑤连接BIS监测仪，操作如下：

使用酒精棉球反复清洁皮肤，待干后使用BIS^XP^专用四导联电极片(BIS-sensor^XP^)，1号电极正对眉间(距鼻根5 cm左右)，2号电极位于1号和4号之间即眉弓上方，4号电极下缘与右侧眉弓平齐，3号电极与眼球中心连线平齐。

（2）麻醉诱导：

①按药物使用说明沙丁胺醇喷雾30 min后行全麻诱导；

②预给氧时氧流量调至8 L·min^-1^，静脉缓慢依次注射芬太尼3.0 μg·kg^-1^，丙泊酚1.5～2.0 mg·kg^-1^，爱可松0.9 mg·kg^-1^；

③气管插管后固定气管导管（女性患者ID 7.0，男性患者ID 8.0），机械控制通气，调节潮气量8～10 mL·kg^-1^，呼吸频率10 bpm，吸呼比1：2，维持EtCO_2_于30～35 mmHg，待血流动力学稳定4～5 min后，开启七氟烷挥发罐（开启挥发罐时采用2%的七氟烷进行回路预充，确保回路七氟烷充填均匀），吸入浓度为2%，氧流量2 L·min^-1^，用旁流法连接气体采样管于气管导管Y型接口一端，采集七氟烷浓度；洗入阶段设定吸入浓度为2%，氧流量2 L·min^-1^， 洗出阶段氧流量2 L·min^-1^。

（3）麻醉维持：

观察期间不给患者任何外源性刺激（包括摆体位），不追加任何麻醉药物；观察期间若出现严重血压下降时（MAP﹤50 mmHg）静注麻黄碱5～10 mg，效果不佳时改用盐酸去氧肾上腺素100～200 μg；心率(HR)减慢(﹤45次·min^-1^)静注阿托品0.25～0.5 mg。

（4）麻醉结束前30 min 通过呼吸回路再次给予沙丁胺醇200μg，术毕前15min关闭挥发罐。

3.4 观察指标

（1）入室时基础心率（HR），有创血压（IBP），脉搏氧饱和度（SpO_2_），测定动脉血气。

（2）挥发罐开启1 min、2 min、3 min、5 min、7 min、10 min和15 min的心率（HR）、有创血压（IBP）、脉搏氧饱和度（SpO_2_），呼气末CO_2_分压（EtCO_2_），吸入七氟烷浓度（Insevo）%和呼气末七氟烷浓度（Etsevo）%，记录患者在气管插管后1 min、5 min、10 min气道峰压(Ppeak)和气道平台压(Pplat)。于一定时间点抽取动脉血进行血气分析。记录复苏期间患者睁眼、拔管、口述生日即刻等时间。

（3）观察术后早期7天内所有患者肺部并发症的发生情况。

3.5 统计学处理和分析

全部试验完成后揭盲，根据药剂编号，将试验者分成沙丁胺醇组和空白对照组进行数据统计分析。采用Stata7.0软件包进行统计分析，所有计量数据均采用均数±标准差（mean±SD）表示，组间差异采用完全随机设计两样本t检验，组内差异采用单因素方差分析（ANOVA，S-N-K方法），计数资料比较采用卡方检验或Fisher确切概率计算，*P*﹤0.05认为差异有统计学意义。

5.2技术路线

符合纳入标准择期进行外科手术COPD患者80例

喷雾剂诱导前30min给予

麻醉诱导

数据采集

外科手术麻醉维持

喷雾剂手术结束前30min给予

麻醉苏醒

数据采集

完成所有病例后揭盲和分组

试验组(沙丁胺醇喷雾)

对照组(空白对照组)

统计学处理

阐明围手术期使用沙丁胺醇可增加肺泡-毛细血管的有效面积，麻醉诱导期间可加速吸入麻醉药物到达肺泡，使麻醉深度用于调控；苏醒期间可加速药物经肺排除，提高苏醒质量，减少呼吸抑制和不良时间发生；降低早期肺部并发症的发生

附：COPD患者诊断标准：COPD诊断主要依据吸烟等高危病史、临床症状、体征及肺功能等检查综合分析确定。长期咳嗽、咳痰病史；桶状胸，胸部叩诊呈过清音，呼气时间延长；胸片显示肺气肿征象；不完全可逆的气流受限是COPD诊断的必要条件；吸入支气管扩张药后FVC＜70%以及FEV_l_＜80%预计值，可以确定为不完全可逆气流受限。支气管扩张试验阳性：FEV_l_增加值≥12%，且△FEV_l_≥200mL。

PPC的判定标准：组成部分包括肺部炎症、呼吸衰竭、胸腔积液、肺不张、气胸、支气管痉挛、吸入性肺炎

**Definitions of postoperative pulmonary complications**

***Respiratory infection***

Treatment with antibiotics for a respiratory infection, plus at least one of the following criteria: new or changed sputum, new or

changed lung opacities, fever, and leukocyte count >12,000/mm3

***Respiratory failure***

Postoperative PaO2 <60mmHg on room air, a ratio of PaO2 to inspired oxygen fraction <300, or SaO2 <90% and requiring oxygen

therapy

***Pleural effusion***

Chest radiograph demonstrating blunting of the costophrenic angle, evidence of displacement of adjacent anatomical structures, or

(in supine position) a hazy opacity in one hemithorax with preserved vascular shadows

***Atelectasis***

Collapse of the alveoli, lung opacification with shift of the mediastinum, hilum, or hemidiaphragm toward the affected area, and

compensatory overinflation in the adjacent nonatelectatic lung

***Pneumothorax***

A collection of air in the pleural space (the area with no vascular bed surrounding the visceral pleura)

***Bronchospasm***

Newly detected expiratory wheezing treated with bronchodilators

***Aspiration pneumonitis***

Acute lung injury after the inhalation of regurgitated gastric contents

PaO2: partial pressure of oxygen in arterial blood; SaO2: arterial oxyhemoglobin saturation.

参考文献：

(1) Downs CA, Appel SA. Chronic obstructive pulmonary disease: Diagnosis and management. Am Acad Nurse Pract, 2007,19: 126-132.

(2) Burrowes KS, De Backer J, Smallwood R, et al.[Multi-scale computational models of the airways to unravel the pathophysiological mechanisms in asthma and chronic obstructive pulmonary disease (AirPROM).](http://www.ncbi.nlm.nih.gov/pubmed/24427517) Interface Focus,2013,3(2):20120057.

(3) Zoeckler N, Kenn K, Kuehl K, et al.[Illness perceptions predict exercise capacity and psychological well-being after pulmonary rehabilitation in COPD patients.](http://www.ncbi.nlm.nih.gov/pubmed/24439691)

J Psychosom Res, 2014 ,76(2):146-151.

(4) Wu Y, Liu F, Tang H, et al.[The analgesic efficacy of subcostal transversus abdominis plane block compared withthoracic epidural analgesia and intravenous opioid analgesia after radical gastrectomy.](http://www.ncbi.nlm.nih.gov/pubmed/23744953)Anesth Analg,2013, 117(2):507-513.

(5)Agzarian J, Miller JD, Kosa SD, et al. [Long-term survival analysis of the Canadian Lung Volume Reduction Surgery trial.](http://www.ncbi.nlm.nih.gov/pubmed/23895890)Ann Thorac Surg,2013,96(4): 1217-1222.

(6)Peduto  VA ,  Mezzetti D , Properzi  M , et al.Sevoflurane provides better recovery than propofol plus fentanyl in anaesthesia for day-care surgery. Eur J Anaesthesiol ,2008,17:138-143.

(7)Hovens IB, Schoemaker RG, van der Zee EA, et al.[Thinking through postoperative cognitive dysfunction: How to bridge the gap between clinical and pre-clinical perspectives.](http://www.ncbi.nlm.nih.gov/pubmed/22728316)Brain Behav Immun, 2012,26(7):1169-1179.

(8) Peyton PJ,Fortuin M,Robinson GB, et al. The rate of alveolar-capillary uptake of sevoflurane and nitrous oxide following anaesthetic induction .Anaesthesia, 2008, 63: 358-363.

(9)Zulkarneev R, Zagidullin N, Abdrahmanova G, et al.[Ivabradine prevents heart rate acceleration in patients with chronic obstructive pulmonary disease and coronary heart disease after salbutamol inhalation.](http://www.ncbi.nlm.nih.gov/pubmed/24281409) Pharmaceuticals (Basel),2012 ,5(4):

398-404.

(10)Tantucci C， Duguet A， Similowski T，et al.Effect of salbutamol on dynamic hyperinflation in chronic obstructive pulmonary disease patients. Eur Respir J，2010, 12: 799-804.

(11)Ross Kennedy R, French RA, Spencer C. Predictive Accuracy of a Model of Volatile Anesthetic Uptake. Anesth Analg 2002;95:1616–1621.

(12)La Piana GE, Corda L, Bertella E, et al.[Dose-response curve to salbutamol during acute and chronic treatment with formoterol inCOPD.](http://www.ncbi.nlm.nih.gov/pubmed/21857779)I nt J Chron Obstruct Pulmon Dis,2011,6:399-405.

(13)Cazzola M, Rogliani P, Ruggeri P, Set al.[Chronic treatment with indacaterol and airway response to salbutamol in stable COPD.](http://www.ncbi.nlm.nih.gov/pubmed/23490225)Respir Med. 2013 Jun;107(6):848-853.

(14)De Backer LA, Vos WG, Salgado R,et al. [Functional imaging using computer methods to compare the effect of salbutamol and ipratropium bromide in patient-specific airway models of COPD.](http://www.ncbi.nlm.nih.gov/pubmed/22162649)Int J Chron Obstruct Pulmon Dis,2011;6(2):637-646.

(15) Patman S. Preoperative physiotherapy education prevented postoperative pulmonary complications following open upper abdominal surgery. BMJ Evid Based Med. 2019 ;24(2):74-75.

(16) Nijbroek SG, Schultz MJ, Hemmes SNT. Prediction of postoperative pulmonary complications. Curr Opin Anaesthesiol. 2019;32(3):443-451.

(17)Miskovic A, Lumb AB. Postoperative pulmonary complications. Br J Anaesth. 2017 1;118(3):317-334.
