# Supplementary material for: Effects of salbutamol on the kinetics of sevoflurane and the occurrence of early postoperative pulmonary complications in patients with mild-to-moderate chronic obstructive pulmonary disease: A randomized controlled study
Source: PLoS One. 2021 May 20;16(5):e0251795. doi: 10.1371/journal.pone.0251795 (PMC8136676; doi:10.1371/journal.pone.0251795)
Supplement: S5 File — (PDF) [file pone.0251795.s009.pdf]

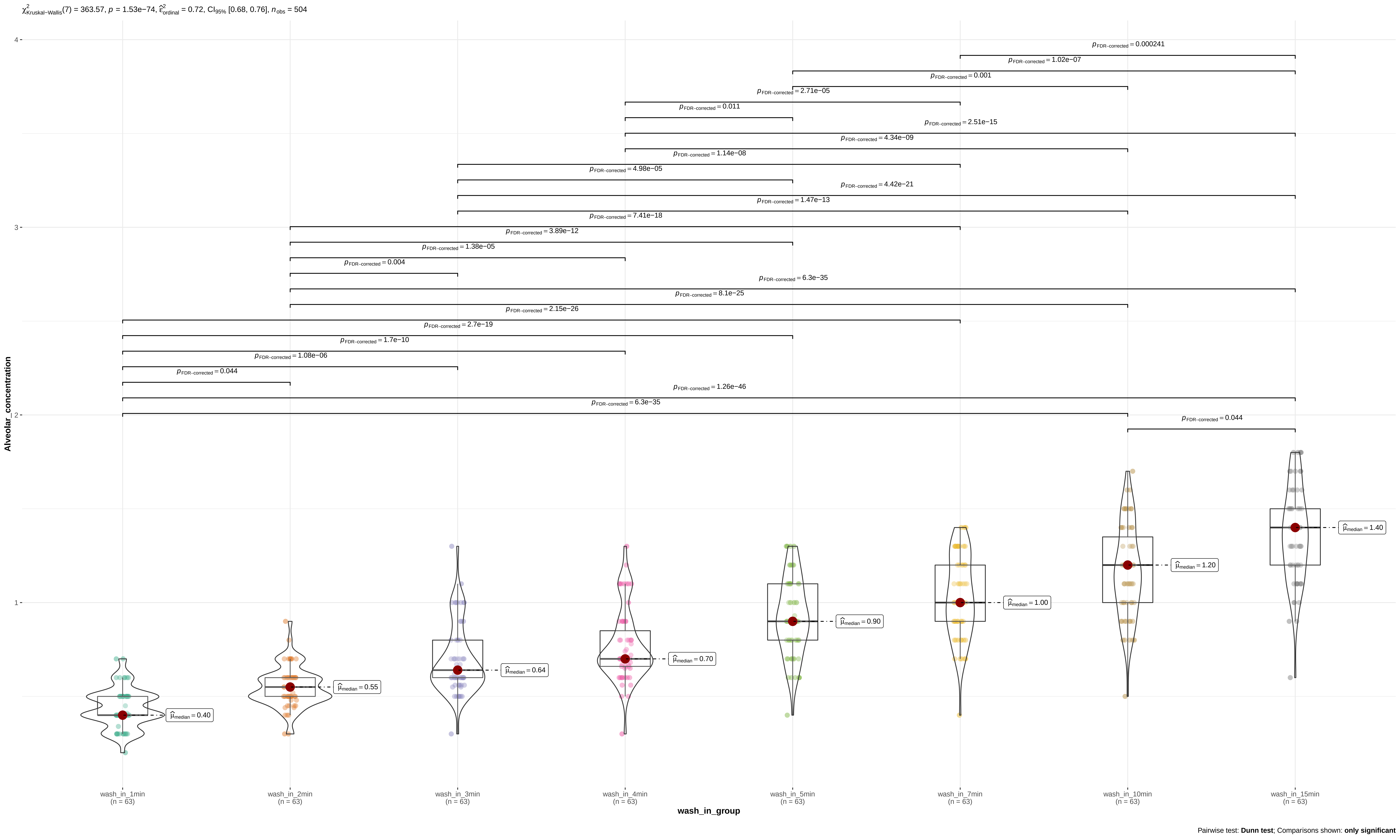
$\chi^2_{Kruskal-Wallis}(7) = 363.57$, $p = 1.53e{-}74$, $\hat{\epsilon}^2_{ordinal} = 0.72$, $CI_{95\%}$ [0.68, 0.76], $n_{obs} = 504$
$p_{FDR-corrected} = 0.000241$
$p_{FDR-corrected} = 1.02e{-}07$
$p_{FDR-corrected} = 0.001$
$p_{FDR-corrected} = 2.71e{-}05$
$p_{FDR-corrected} = 0.011$
$p_{FDR-corrected} = 2.51e{-}15$
$p_{FDR-corrected} = 4.34e{-}09$
$p_{FDR-corrected} = 1.14e{-}08$
$p_{FDR-corrected} = 4.98e{-}05$
$p_{FDR-corrected} = 4.42e{-}21$
$p_{FDR-corrected} = 1.47e{-}13$
$p_{FDR-corrected} = 7.41e{-}18$
$p_{FDR-corrected} = 3.89e{-}12$
$p_{FDR-corrected} = 1.38e{-}05$
$p_{FDR-corrected} = 0.004$
$p_{FDR-corrected} = 6.3e{-}35$
$p_{FDR-corrected} = 8.1e{-}25$
$p_{FDR-corrected} = 2.15e{-}26$
$p_{FDR-corrected} = 2.7e{-}19$
$p_{FDR-corrected} = 1.7e{-}10$
$p_{FDR-corrected} = 1.08e{-}06$
$p_{FDR-corrected} = 0.044$
$p_{FDR-corrected} = 1.26e{-}46$
$p_{FDR-corrected} = 6.3e{-}35$
$p_{FDR-corrected} = 0.044$
Alveolar_concentration
4
3
2
1
$\hat{\mu}_{median} = 0.40$
$\hat{\mu}_{median} = 0.55$
$\hat{\mu}_{median} = 0.64$
$\hat{\mu}_{median} = 0.70$
$\hat{\mu}_{median} = 0.90$
$\hat{\mu}_{median} = 1.00$
$\hat{\mu}_{median} = 1.20$
$\hat{\mu}_{median} = 1.40$
wash_in_1min (n = 63)
wash_in_2min (n = 63)
wash_in_3min (n = 63)
wash_in_4min (n = 63)
wash_in_5min (n = 63)
wash_in_7min (n = 63)
wash_in_10min (n = 63)
wash_in_15min (n = 63)
wash_in_group
Pairwise test: Dunn test; Comparisons shown: only significant
