# Supplementary material for: Effects of salbutamol on the kinetics of sevoflurane and the occurrence of early postoperative pulmonary complications in patients with mild-to-moderate chronic obstructive pulmonary disease: A randomized controlled study
Source: PLoS One. 2021 May 20;16(5):e0251795. doi: 10.1371/journal.pone.0251795 (PMC8136676; doi:10.1371/journal.pone.0251795)
Supplement: S6 File — (PDF) [file pone.0251795.s010.pdf]

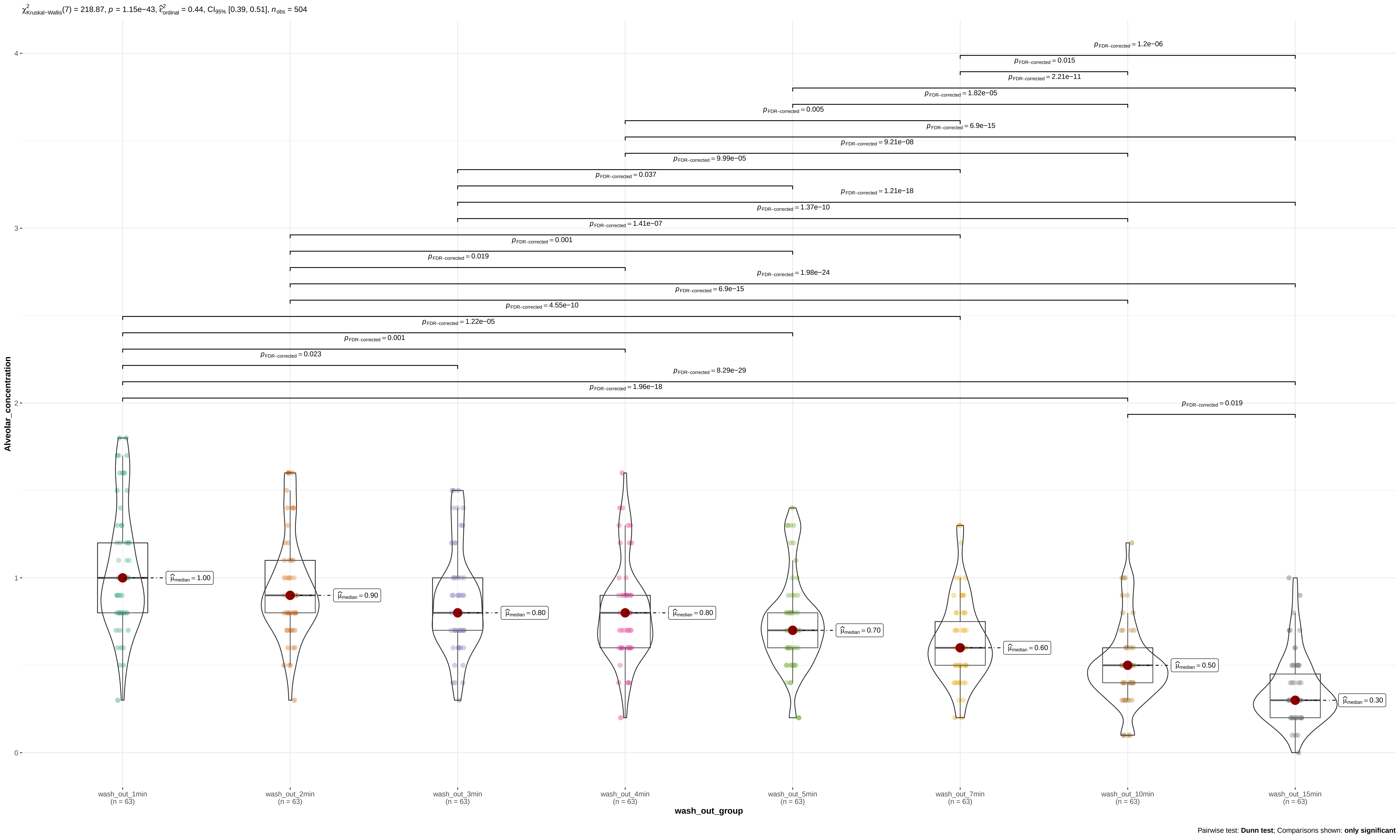
$\chi^2_{\text{Kruskal-Wallis}}(7) = 218.87$, $p = 1.15e-43$, $\hat{\varepsilon}^2_{\text{ordinal}} = 0.44$, $\text{CI}_{95\%}$ [0.39, 0.51], $n_{\text{obs}} = 504$
$p_{\text{FDR-corrected}} = 1.2e-06$
$p_{\text{FDR-corrected}} = 0.015$
$p_{\text{FDR-corrected}} = 2.21e-11$
$p_{\text{FDR-corrected}} = 1.82e-05$
$p_{\text{FDR-corrected}} = 0.005$
$p_{\text{FDR-corrected}} = 6.9e-15$
$p_{\text{FDR-corrected}} = 9.21e-08$
$p_{\text{FDR-corrected}} = 9.99e-05$
$p_{\text{FDR-corrected}} = 0.037$
$p_{\text{FDR-corrected}} = 1.21e-18$
$p_{\text{FDR-corrected}} = 1.37e-10$
$p_{\text{FDR-corrected}} = 1.41e-07$
$p_{\text{FDR-corrected}} = 0.001$
$p_{\text{FDR-corrected}} = 0.019$
$p_{\text{FDR-corrected}} = 1.98e-24$
$p_{\text{FDR-corrected}} = 6.9e-15$
$p_{\text{FDR-corrected}} = 4.55e-10$
$p_{\text{FDR-corrected}} = 1.22e-05$
$p_{\text{FDR-corrected}} = 0.001$
$p_{\text{FDR-corrected}} = 0.023$
$p_{\text{FDR-corrected}} = 8.29e-29$
$p_{\text{FDR-corrected}} = 1.96e-18$
$p_{\text{FDR-corrected}} = 0.019$
Alveolar_concentration
4
3
2
1
0
$\hat{\mu}_{\text{median}} = 1.00$
$\hat{\mu}_{\text{median}} = 0.90$
$\hat{\mu}_{\text{median}} = 0.80$
$\hat{\mu}_{\text{median}} = 0.80$
$\hat{\mu}_{\text{median}} = 0.70$
$\hat{\mu}_{\text{median}} = 0.60$
$\hat{\mu}_{\text{median}} = 0.50$
$\hat{\mu}_{\text{median}} = 0.30$
wash_out_1min (n = 63)
wash_out_2min (n = 63)
wash_out_3min (n = 63)
wash_out_4min (n = 63)
wash_out_5min (n = 63)
wash_out_7min (n = 63)
wash_out_10min (n = 63)
wash_out_15min (n = 63)
wash_out_group
Pairwise test: **Dunn test**; Comparisons shown: **only significant**
