# Supplementary material for: Effects of salbutamol on the kinetics of sevoflurane and the occurrence of early postoperative pulmonary complications in patients with mild-to-moderate chronic obstructive pulmonary disease: A randomized controlled study
Source: PLoS One. 2021 May 20;16(5):e0251795. doi: 10.1371/journal.pone.0251795 (PMC8136676; doi:10.1371/journal.pone.0251795)
Supplement: Attachment — Submitted filename: renamed_27e72.docx [file pone.0251795.s011.docx]

Dear editor,

Thank you for your work.

We have revised the manuscript point by point according to the reviewers’ opinion, some paragraphs have been totally rewritten. Few questions we rebut and discuss the related points in the response.

The follows exactly showed that points(we did not change or delete the original versions you sent to us):

Thank you for your consideration. I look forward to hearing from you.

Sincerely,

Dr. Zongming Jiang

Professor and supervisor

Department of Anesthesia

Shaoxing People’s Hospital

No 568,North Zhongxing Road, Yuecheng District of Shaoxing City

Shaoxing, Zhejiang 312000, China

Tel: 86-10-575-88229212

Fax:86-10-575-88229226

E-mail: jiangzhejiang120@163.com

PONE-D-20-33323R1
Effects of salbutamol on the kinetics of sevoflurane and the occurrence of early PPC in patients with mild to moderate COPD: a randomized controlled study
PLOS ONE

Dear Dr. Jiang,

Thank you for submitting your manuscript to PLOS ONE. After careful consideration, we feel that it has merit but does not fully meet PLOS ONE’s publication criteria as it currently stands. Therefore, we invite you to submit a revised version of the manuscript that addresses the points raised during the review process.

Please submit your revised manuscript by Apr 19 2021 11:59PM. If you will need more time than this to complete your revisions, please reply to this message or contact the journal office at plosone@plos.org. When you're ready to submit your revision, log on to https://www.editorialmanager.com/pone/ and select the 'Submissions Needing Revision' folder to locate your manuscript file.

Please include the following items when submitting your revised manuscript:

- A rebuttal letter that responds to each point raised by the academic editor and reviewer(s). You should upload this letter as a separate file labeled 'Response to Reviewers'.
- A marked-up copy of your manuscript that highlights changes made to the original version. You should upload this as a separate file labeled 'Revised Manuscript with Track Changes'.
- An unmarked version of your revised paper without tracked changes. You should upload this as a separate file labeled 'Manuscript'.

If you would like to make changes to your financial disclosure, please include your updated statement in your cover letter. Guidelines for resubmitting your figure files are available below the reviewer comments at the end of this letter.

If applicable, we recommend that you deposit your laboratory protocols in protocols.io to enhance the reproducibility of your results. Protocols.io assigns your protocol its own identifier (DOI) so that it can be cited independently in the future. For instructions see: <http://journals.plos.org/plosone/s/submission-guidelines#loc-laboratory-protocols>

We look forward to receiving your revised manuscript.

Kind regards,

Walid Kamal Abdelbasset, Ph.D.
Academic Editor
PLOS ONE

**Answer:We have uploaded the protocols to protocols.io and DOI is:
dx.doi.org/10.17504/protocols.io.btidnka6**

[Note: HTML markup is below. Please do not edit.]

Reviewers' comments:

Reviewer's Responses to Questions

**Comments to the Author**

1. If the authors have adequately addressed your comments raised in a previous round of review and you feel that this manuscript is now acceptable for publication, you may indicate that here to bypass the “Comments to the Author” section, enter your conflict of interest statement in the “Confidential to Editor” section, and submit your "Accept" recommendation.

Reviewer #1: All comments have been addressed

Reviewer #2: All comments have been addressed

Reviewer #3: (No Response)

2. Is the manuscript technically sound, and do the data support the conclusions?

The manuscript must describe a technically sound piece of scientific research with data that supports the conclusions. Experiments must have been conducted rigorously, with appropriate controls, replication, and sample sizes. The conclusions must be drawn appropriately based on the data presented.

Reviewer #1: Partly

Reviewer #2: Yes

Reviewer #3: Partly

3. Has the statistical analysis been performed appropriately and rigorously?

Reviewer #1: Yes

Reviewer #2: Yes

Reviewer #3: No

4. Have the authors made all data underlying the findings in their manuscript fully available?

The [PLOS Data policy](http://www.plosone.org/static/policies.action#sharing) requires authors to make all data underlying the findings described in their manuscript fully available without restriction, with rare exception (please refer to the Data Availability Statement in the manuscript PDF file). The data should be provided as part of the manuscript or its supporting information, or deposited to a public repository. For example, in addition to summary statistics, the data points behind means, medians and variance measures should be available. If there are restrictions on publicly sharing data—e.g. participant privacy or use of data from a third party—those must be specified.

Reviewer #1: Yes

Reviewer #2: No

Reviewer #3: No

5. Is the manuscript presented in an intelligible fashion and written in standard English?

PLOS ONE does not copyedit accepted manuscripts, so the language in submitted articles must be clear, correct, and unambiguous. Any typographical or grammatical errors should be corrected at revision, so please note any specific errors here.

Reviewer #1: No

Reviewer #2: Yes

Reviewer #3: (No Response)

6. Review Comments to the Author

Please use the space provided to explain your answers to the questions above. You may also include additional comments for the author, including concerns about dual publication, research ethics, or publication ethics. (Please upload your review as an attachment if it exceeds 20,000 characters)

Reviewer #1: Reviewer comments:
Thank you for giving the opportunity to review this article.
Abstract:
1. Include the acronym of abbreviation when using first time.

Answer: It has been changed like this:chronic obstructive pulmonary disease (COPD) patients.
Introduction
2. Please check the grammar, syntax and paragraph format.

Answer: we have re-formatted and check the gramma.
3. Add the clinical significance of this article over the participants and researchers.

Answer: The significance of our current study is that the results of the study will provide evidence for treating patients with COPD as regards to kinetics of volatiles and pulmonary complications in clinical practice.
Methods
4. Include the study design, randomization and allocation procedure.

Answer: Patients were randomized in a 1:1 ratio to either the salbutamol group or the control group. Randomization was computer-generated and each patient was given a unique randomization number (patient code).The block length was four. The investigators who were responsible for assessing the primary endpoints, as well as the anesthesiologists, postoperative care unit nursing staff, and variable assessors, were blinded to study group assignment. The staff members who collected data during surgery were aware of the group assignments. The salbutamol preparations and its identical in appearance placebo (normal saline) were used to minimize the bias in this study. Salbutamol aerosol inhalers and its identical placebo (containing normal saline) were consecutively numbered from 1 to 80. And the eligible patients were also numbered based on sequence enrolled into the study.
5. Include the reliability and validity of outcome measures with references.

Answer: The outcome measures : The F_A_/F_I_ ratio was assessed to represent the processing of sevoflurane. F_A_ and F_I_ are extracted from the anesthesia machine, which has module to monitor and measure. Before each experiment, we will calibrate it according to the instructions. It is reliable. Determinations of other parameters were based on the standard definitions, for example PPC.
Results
6. Include the reports with CI 95% with p scores..

Answer: It has been analyzed and added.
7. Mention the effect size of primary variable and its MCID scores.

Answer: Bronchodilators produce bronchodilation and increase lung volumes, improves volume bulk movement in patients withCOPD. Effect size in our study is the fraction difference of sevoflurane in the admixture of inspired gas, the clinical significance is reflecting the wash-in of volatiles, a faster wash-in produces shorter time to the desired depth of anesthesia. If in awake patients, there will be difference in time of loss of consciousness. The MCID is difficult to determine, and often based on experts’ opinion or clinical experience in the current study. According to the literature:Torri G, Casati A, Comotti L, Bignami E, Santorsola R, Scarioni M. Wash-in and wash-out curves of sevoflurane and isoflurane in morbidly obese patients. Minerva Anestesiol. 2002 Jun;68(6):523-7. PMID: 12105408. The minimal clinically important difference(**MCID**) is 0.05 to 0.08 of exhaled sevoflurane concentration, which often produce a little faster time of loss of consciousness (15-22seconds).In fact ,it is a complicated interplay between respiration and cardiovascular system. So, we did not clearly point out the effect size of primary variable and its MCID scores in the manuscript. As in the paper :Sharma P, Gombar S, Ahuja V, Jain A, Dalal U. Sevoflurane sparing effect of dexmedetomidine in patients undergoing laparoscopic cholecystectomy: A randomized controlled trial. J Anaesthesiol Clin Pharmacol. 2017 Oct-Dec;33(4):496-502. doi: 10.4103/joacp.JOACP_144_16. PMID: 29416243; PMCID: PMC5791264.

Discussion
8. The discussion part should discuss the relation between the outcome variables with latest references.

Answer:We have related the current results to the previous studies.
9. Include the future recommendations of this study.

Answer: we recommend like this as the followings: Despite the results achieved in the study, we recommended that further studies should be conducted to testify the results and its applications. As follows: (1) the time and optimal dose for inhaling nebulized salbutamol perioperatively is further needed to clarify the effects on respiratory mechanics; (2) whether the use of nebulized salbutamol decreases the PPC occurrence in patients with severe COPD.(3) whether lung protective ventilation in conjunction with salbutamol aerosol confers beneficial effect on PPC during general anesthesia.

Reviewer #2: The authors have essentially dealt with my substantive criticisms of their previous statistical analysis. Even though we might disagree on some minor issues, the choices made by the authors are reasonable and allow the readers to make their own determination if they disagree a little.

My primary current reservations around the manuscript center around the English language usage. There are many small errors in grammar and usage that should probably be addressed, especially in the newly minted statistical methods section.

Answer: Language had been proof through technical assistance.

Reviewer #3: Authors partly revised the Ms. and some of the suggestions were not addressed.
Authors did not mention how they reached the sample size in the methods

Answer: We draw the sample size as follows: After careful search of the literature on the internet, we found out that no similar study pertaining to the kinetics of sevoflurane in COPD patients undergoing elective surgery and so a pilot pretest study was conducted (6 cases in each group) to detect the number of subjects required in the study. In the presence of salbutamol aerosol inhalation 30 minutes before anesthesia induction would produce a difference of 0.15±0.03 in end-tidal concentration of sevoflurane. Using a power of 95% and a two-tailed significance level of 0.05, we calculated that a sample size of 25 patients was required. Accounting for a 10% dropout rate due to loss of follow-up or incomplete data, we intended to enroll 30 patients in each group.
Authors need to transparently mention the stst analyses method and type of data in each illustration (in footnotes mention the type of data presented and stat test)

Answer: we added and stated the detailed analyses and footnotes.
In methods:the difference at each time point were determined by Student’s t-tes (this is not correct to be done, t test is not the correcct test for the situation, all values should be assessed by ANOVA then a post hoc test)
& nothing is mentioned in the footnotes!!
Authors wrote also: were compared by the student t-test or the Mann-Whitney test. : this reviewer feels different from that wrote in the table footnote , what are the basis for selecting any of the 2 tests???
Answer: The study had 2 groups. First, we do the homogeneity of variance between 2 groups. Then decided to perform student t-test or the Mann-Whitney test.
Overall the stat analysis in METHODs is not matched with that in the tables. Footnotes does not include sufficient info about the data
Answer: the footnotes have been added and revised in the tables.
Authors wrote: Pearson Chi-square test was used to compare difference between two groups.
Q: Is there a test called {Pearson Chi-square test}?????

Answer: Be honest. This is the error, we already revise it.

7. PLOS authors have the option to publish the peer review history of their article ([what does this mean?](https://journals.plos.org/plosone/s/editorial-and-peer-review-process#loc-peer-review-history)). If published, this will include your full peer review and any attached files.


If you choose “no”, your identity will remain anonymous but your review may still be made public.


**Do you want your identity to be public for this peer review?** For information about this choice, including consent withdrawal, please see our [Privacy Policy](https://www.plos.org/privacy-policy).

Reviewer #1: **Yes:** Gopal Nambi

Reviewer #2: No

Reviewer #3: No

[NOTE: If reviewer comments were submitted as an attachment file, they will be attached to this email and accessible via the submission site. Please log into your account, locate the manuscript record, and check for the action link "View Attachments". If this link does not appear, there are no attachment files.]

While revising your submission, please upload your figure files to the Preflight Analysis and Conversion Engine (PACE) digital diagnostic tool, <https://pacev2.apexcovantage.com/>. PACE helps ensure that figures meet PLOS requirements. To use PACE, you must first register as a user. Registration is free. Then, login and navigate to the UPLOAD tab, where you will find detailed instructions on how to use the tool. If you encounter any issues or have any questions when using PACE, please email PLOS at [figures@plos.org](mailto:figures@plos.org). Please note that Supporting Information files do not need this step.
